# Bayesian Integration of Information in Hippocampal Place Cells

**DOI:** 10.1371/journal.pone.0089762

**Published:** 2014-03-06

**Authors:** Tamas Madl, Stan Franklin, Ke Chen, Daniela Montaldi, Robert Trappl

**Affiliations:** 1 School of Computer Science, University of Manchester, Manchester, United Kingdom; 2 Institute for Intelligent Systems, University of Memphis, Memphis, Tennessee, United States of America; 3 School of Psychological Sciences, University of Manchester, Manchester, United Kingdom; 4 Austrian Research Institute for Artificial Intelligence, Vienna, Austria; University College of London - Institute of Neurology, United Kingdom

## Abstract

Accurate spatial localization requires a mechanism that corrects for errors, which might arise from inaccurate sensory information or neuronal noise. In this paper, we propose that Hippocampal place cells might implement such an error correction mechanism by integrating different sources of information in an approximately Bayes-optimal fashion. We compare the predictions of our model with physiological data from rats. Our results suggest that useful predictions regarding the firing fields of place cells can be made based on a single underlying principle, Bayesian cue integration, and that such predictions are possible using a remarkably small number of model parameters.

## Introduction

For successful navigation, an organism needs to be able to localize itself (i.e. determine its position and orientation) as well as its goal, and it needs to be able to calculate a route between these locations. Since the first reports of physiological evidence for hippocampal ‘place cells’ [1] which exhibit increased firing only in specific locations in the environment, there have been a large number of empirical findings supporting the idea that the Hippocampal-Entorhinal Complex (HEC) is a major neuronal correlate underlying spatial localization and mapping [2].

To keep track of their location when they move, mammals must integrate self-motion signals, and use them to update their location estimate, using a process commonly referred to as path integration or dead reckoning. It has been suggested that self-motion information might be the primary constituent in the formation of the firing fields of place cells [3], [4]. However, path integration alone is prone to accumulating errors (arising from the inaccuracy of sensory inputs and neuronal noise), which add up over time until the location estimate becomes too inaccurate to allow for efficient navigation [5], [6]. Because path integration errors are cumulative, path integrators have to be corrected using allothetic sensory information from the environment in order to ensure that the estimated location will stay close to the true location.

It has also been suggested that place cells rely heavily on visual information [1], [2], [7]. However, the question of how exactly different sources of information are combined, from different boundaries or landmarks, has received little attention in the literature. This paper investigates how place cells in the Hippocampus might integrate information to provide an accurate location estimate. We propose that the integration of cues from different sources might occur in an approximately Bayesian fashion; i.e. that the information is weighted according to its accuracy when combined with a final estimate, with more precise information receiving a higher importance weight. We provide supporting evidence and theoretical arguments for this claim in the Results section. We will compare neuronal recordings of place cells with predictions of a Bayesian model, and present a possible explanation for how approximate Bayesian inference, although insufficient to fully explain firing fields, might provide a useful framework within which to understand cue integration. Finally, we will present a possible model of how Bayesian inference might be implemented at the neuronal level in the hippocampus.

Our results are consistent with the ‘Bayesian brain hypothesis’ [8]; the idea that the brain integrates information in a statistically optimal fashion. There is increasing behavioural evidence for Bayesian informational integration for different modalities, e.g. for visual and haptic [9], for force [10], but also for spatial information, e.g. [11] (see Discussion). Other models of statistically optimal or near-optimal spatial cue integration have been proposed previously [11]–[14], although mostly at Marr's computational or algorithmic level, rather than at a physical level. The latter, mechanistic Bayesian view, has been cautioned against due to lacking evidence on the single neuron level [15]. Our results partially account for three disparate single-cell electrophysiological data sets using a Bayesian framework, and suggest that although such models might be too simple to fully explain patterns of neuronal firing, they will still be highly valuable to our understanding of the relationship between neuronal activity and the environment.

### Neuronal correlates of localization

Here we briefly summarize the neuroscientific literature concerning how mammalian brains represent space. Most of these results come from animal (rat, and to a lesser extent, monkey) cellular recording studies, although there is some recent evidence substantiating the existence of these cell types in humans.

Four types of cells play an important role for allocentric spatial representations in mammalian brains:

1. **Grid cells** in the medial entorhinal cortex show increased firing at multiple locations, regularly positioned in a grid across the environment consisting of equilateral triangles [16]. Grids from neighbouring cells share the same orientation, but have different and randomly distributed offsets, meaning that a small number of them can cover an entire environment. It has also been suggested that grid cells play a major role in path integration, their activation being updated depending on the animal's movement speed and direction [2], [16]–[18]. There is evidence to suggest that they exist not only in mammals, but also in the human entorhinal cortex (EC) [19].
2. **Head-direction cells** fire whenever the animal's head is pointing in a certain direction. The primary circuit responsible for head direction signals projects from the dorsal tegmental nucleus to the lateral mammillary nucleus, anterior thalamus and postsubiculum, terminating in the entorhinal cortex [20]. There is evidence that head direction cells exist in the human brain within the medial parietal cortex [21].
3. **Border cells** and **boundary vector cells** (BVCs), which are cells with boundary related firing properties. The former [22], [23] seem to fire in proximity to environment boundaries, while the firing of the latter [2], [24] depends on boundary proximity as well as direction relative to the mammal's head. Cells with these properties have been found in the mammalian subiculum and entorhinal cortex [22], [23], and there is also some behavioural evidence substantiating their existence in humans [24].
4. **Place cells** are pyramidal cells in the hippocampus which exhibit strongly increased firing in specific spatial locations, largely independent from orientation in open environments [2], [25], thus providing a representation of an animal's (or human's [26]) location in the environment. A possible explanation for the formation of place fields (the areas of the environment in which place cells show increased firing) is that they emerge from a combination of grid cell inputs on different scales [3], [4]. It has also been proposed that place fields might be mainly driven by environmental geometry, arising from a sum of boundary vector cell inputs [7], [24]. This model has successfully accounted for a number of empirical observations, e.g. the effects of environment deformations [7], or of inserting a barrier into an environment, on place fields [24].

Hippocampal place cells play a prominent role in navigation, the association of episodic memories with places, and other important spatio-cognitive functions, which might be impaired if their place fields were inaccurate. However, neither of the outlined place field models fully explain how place cells combine different inputs for accurate localization. The grid cell input model is subject to corruption of the location estimate by accumulating errors which would eventually render the estimate useless unless corrected by observations (see Introduction). On the other hand, boundary vectors alone (if driven solely by geometry, not by features) do not always yield unambiguous location estimates [14]. Even given complex visual information (which border-related cells do not seem to respond to [22], and of which a rat might not see much, given its poor visual acuity [27]), localization without path integration is difficult (localization without odometry was solved in robotics only recently, and is still much more error-prone than combining observations with odometry [28]). For many place cells, both the path integration inputs from grid cells and observation inputs from border-related cells (and possibly others) seem to be required in order to ensure accuracy and certainty. This has been pointed out before (e.g. [29]), but the question of how exactly these inputs are combined has received little attention (but see the Discussion section for related work).

A further, as of yet unanswered, question is how exactly information from different sources (boundaries, landmarks, different senses etc.) might be combined. Although the BVC model made detailed predictions as to the kinds of inputs received by place cells, was fitted successfully to electrophysiological data, and matched empirical observations (such as what happens with place fields on barrier insertion), it does not propose a general principle of cue integration. In order for the model to accurately reflect place field location and size in a given environment, a number of weight and tuning parameters have to be adjusted for every single place cell [7], [24]. In contrast, the Bayesian hypothesis that we investigate in this work implies a general underlying principle for how inputs into place cells are weighted; according to their precision and with more accurate inputs influencing the result stronger than less accurate inputs. The biggest advantage of such a general principle is that it significantly reduces the number of parameters required to account for large datasets (see Results).

Please note that we adopt a highly simplified and constrained view of HEC function and anatomy in this paper. Hippocampal cells play a role in many cognitive functions other than spatial localization; among others long-term episodic/declarative memory [30], [31], memory based prediction [32], and possibly short-term memory [33] and perception [34]. Furthermore, place cells receive a broader array of inputs than just those transmitting visual and path integration information, such as odours and tactile information [35]. Finally, while cells from different parts of the hippocampus differ in their connectivity and in the information they receive, we believe that dealing with a small subset of functionality and anatomy suffices for investigating the existence of statistically near-optimal information integration in place cells.

### Hypotheses

In this paper, we describe a Bayesian mechanism of information integration in place cells accounting for place field formation. This mechanism rests on the following hypotheses:

**H1**. Some Hippocampal place cells perform approximate Bayesian cue integration - they combine different sources of information in an approximately Bayes-optimal fashion, weighting inputs according to their precision. This means that when sensory inputs change, some place fields should shift and resize in a manner predictable by a Bayesian model.
**H2**. A Bayesian view requires that HEC neurons encode a mammal's uncertainty regarding its position, in addition to its actual location. We hypothesize that the sizes of place cell firing fields are correlated with this location uncertainty.
**H3**. The uncertainty of distance measurements to borders 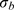 depends on the boundary distance 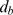, and can be approximated by a linear relationship using some constant 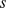 (cf. Weber's law): 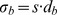. There is some physiological evidence for this in border-related cells [22], [23], as well as some behavioural evidence that Weber's law holds for spatial distance perception in rats [36] and mammals [37]. That the tuning breadths of BVCs should increase with distance is also a prerequisite of the Boundary Vector Cell model [7], [24], has been successfully fit to neuronal and behavioural data, and is supported by physiological evidence [22].

These hypotheses are interdependent, and will be investigated together. To generate verifiable predictions from the Bayesian hypothesis (H1) we need to assume how uncertainty is represented (H2) and how it can be derived from the geometry of the environment (H3). Together, these hypotheses allow the making of predictions about the sizes of place cell firing fields, given the distances of all boundaries, in some cases using just a single parameter specifying how uncertainty depends on distance. The Bayesian mechanism attempts to account for the sizes of single firing fields, deriving them from the distances of boundaries or obstacles (H3) - thus, place cells with multiple firing fields can be modelled by dealing with each firing field separately, even under Gaussian assumptions. In the Discussion section, we briefly describe how the model could be extended by relaxing some of its assumptions, and we report applications of the extended model in the Results section. We do not claim that place cells implement any statistical equation (especially not the simplistic ones described here), but we propose that investigating their firing fields within a statistical framework can yield useful insights about the way they combine information.

## Methods

The hypothesis of approximate Bayesian integration of information in place cells (H1) yields verifiable electrophysiological predictions. Since we hypothesized that place cells can perform approximate Bayesian cue integration (H1), and place field sizes are correlated with uncertainty (H2), and that uncertainty depends on distance (H3), expected place field sizes can be predicted from the geometry of an environment using a Bayesian model. This section will outline such a Bayesian model.

### Model assumptions

To simplify the mathematics, and because this assumption fits our data well, we will assume elliptical firing fields shaped like two-dimensional Gaussians. We do not claim that place cells encode exact Gaussian distributions (there are also asymmetric place fields in the hippocampus - see the Discussion for potential extensions of this simple model). However, investigating their firing fields in a Bayesian framework can yield useful insights about cue integration. The predictions in the Results section are generated from Bayesian models using Gaussian probability distributions to represent locations, in simplified two-dimensional environments, with sizes and distances adjusted to those of the respective in-vivo experiments.

### Bayesian spatial cue integration

Bayesian inference under Gaussian assumptions implies that information from different observations should be weighted according to its accuracy. This claim can be formalized using Bayes' rule, according to which the probability distribution of the location given a number of observations can be calculated from
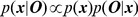
(1)where 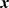 is the animals location in the environment and 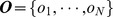 represents a set of 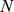 observations. 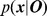 is the posterior location belief, given all observations. 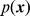 is a prior belief over the location (for example via path integration), and 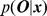 represents the probability of the current observations given 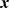 (such as boundaries or landmarks), characterized by the distance from 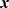 and their uncertainty (see below). Since observations can be assumed to be conditionally independent given the location (this is an assumption commonly made in robotics, see [38], [39]), we can expand equation (1) to
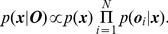
(2)


In this simplified model, the probability distributions are assumed to be Gaussian. Thus, for multiple spatial dimensions, equation (2) can be written as
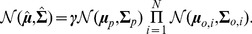
(3)


In the case of a single spatial dimension, and in environments where spatial dimensions can be assumed to be independent and thus can be considered separately, equation (2) can be written as
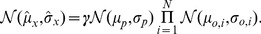
(4)


Here, 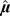 (or 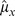 in one dimension) is the mean of the posterior or the ‘best guess’ location, 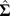 (or 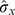 in one dimension) the uncertainty (covariance, or standard deviation) associated with this location, 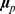 (or 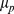) and 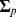 (or 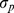) are the mean and the uncertainty of the prior belief location, 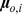 (or 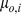) and 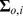 (or 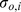) are the means and uncertainties of the individual observations, and 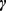 is a constant for normalization.

**Figure 1:**
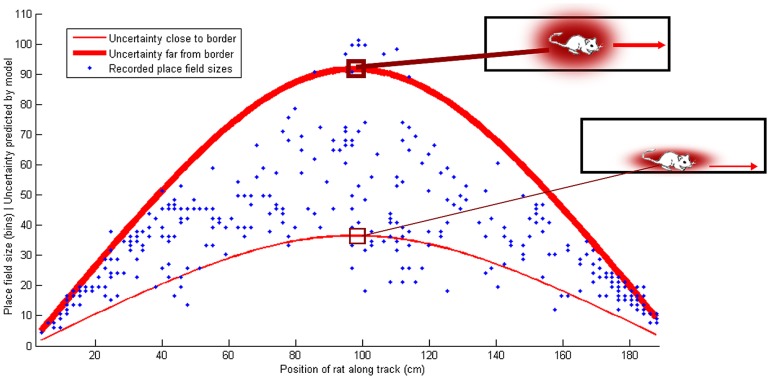
Place field sizes, and predicted uncertainty, on an empty rectangular track. The blue dots show the sizes of individual place fields in bins (one bin equals 1.9 cm). The red lines show the location uncertainty predicted by the Bayesian model - the thin red line (bottom) represents a trajectory very close to either the top or the bottom border (which means a small uncertainty in the 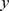 dimension), and the thick red line (top) shows a trajectory in the middle of the track, far from the borders (which means a large uncertainty in the 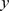 dimension). They account for 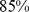 of the place fields between them and thus explain most of the variance. (Data from [68]).

**Figure 2:**
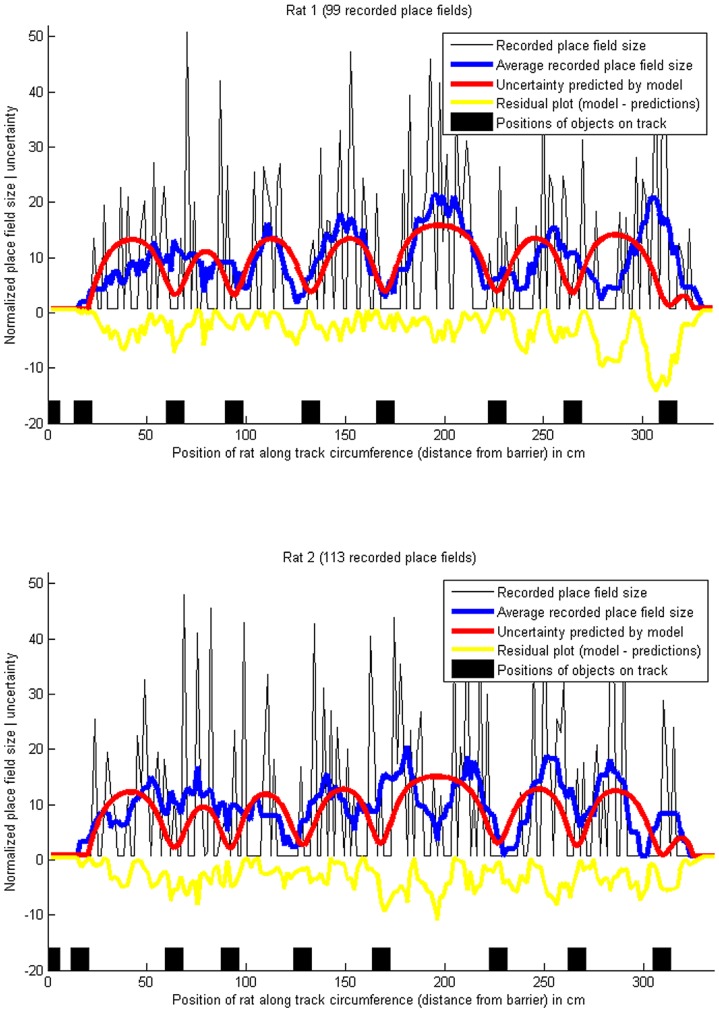
Place field sizes, and predicted uncertainty, on a circular track with objects. The blue lines show the smoothed place field sizes (10-point moving average), normalized to a mean of 0 and variance of 1, and the red lines show the location uncertainty predicted by the Bayesian model. The minima of the red lines correspond to the black squares marking the positions of the objects on the track, since the location uncertainty is lowest near to an object and highest when the rat is far from the objects. Pearson's correlation coefficient between the recorded place field sizes and the predicted uncertainty was 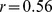 for rat 1 and 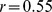 for rat 2. The proportions of explained variance were 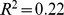 for rat 1 and 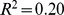 for rat 2. (Data from [42]).

**Figure 3:**
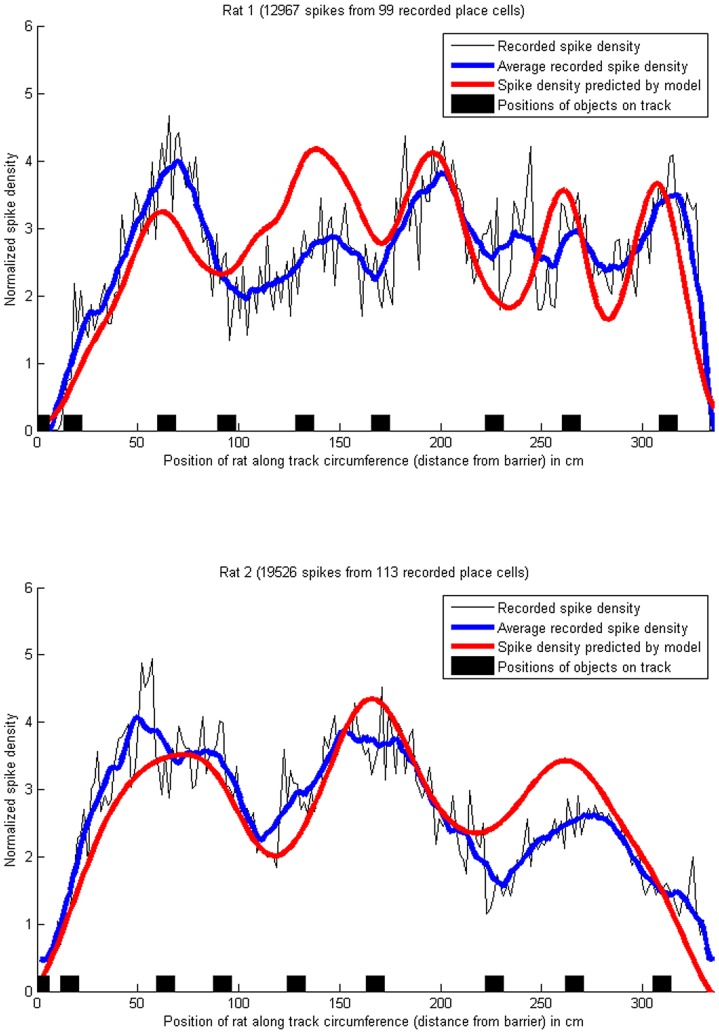
Predicted and recorded place fields in environment B. The squares represent firing rates at each point of the big square environment, with hot colors marking high firing rates, and cold colors low firing rates (the plots have been scaled to fit the page - see main text for the actual proportions of the environments). The model prediction was made based on parameters estimated from the other environments (environments A, C and D). The overall mean proportion of explained variance was 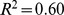 (Data from [69]).

Calculating the uncertainty 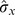 (standard deviation) in one spatial dimension is sometimes sufficient in environments in which the width is negligible compared to the length (such as the first two environments in the Results section: the linear track in Figure 1, and the circular track in Figure 2). In the rectangular environments of Figure 3, the 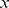 and 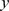 dimensions were assumed to be independent, and the uncertainties were calculated independently - which is a reasonable approximation for this particular dataset, since the observations (the walls of the environment) were orthogonal. However, for more complex environments, the covariances 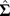 would have to be calculated from equation (3) instead of individually calculating the standard deviations in each dimension (see Text S1 in the Supporting Information for the derivation of the covariance matrix from distance measurements, for two-dimensional environments in which the dimensions cannot be assumed to be independent).

In the one-dimensional case, solving equation (4) for the standard deviations (see [40] for the derivation of the standard deviation of a product of Gaussians), we can calculate the uncertainty associated with the ‘best guess’ location, 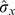, which for a single observation is
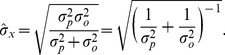
(5)


For 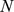 observations, the uncertainty is:
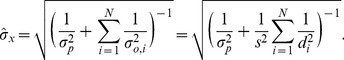
(6)


According to hypothesis 3 (see Hypotheses section), the observation uncertainty is proportional to the distance 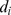: 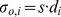. Thus, 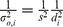. Substituting the precision or accuracy of the prior belief 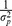 by 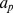, and the factor 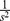 influencing observation precision (i.e. how rapidly the accuracy of distance judgements decreases with increasing distance) by 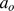, we arrive at equation (7), which can be used to calculate the resulting uncertainty given a prior belief accuracy (which might depend on the path integrator) and the distances and accuracies of all observations.
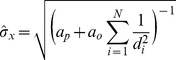
(7)


Equation (7) was used in the Results section to predict uncertainties (hypothesized to be correlated with place field sizes), given distances to boundaries or landmarks. Explained proportions of variance 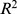 were calculated from 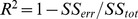, where 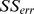 is the sum of squared differences between the model prediction and the recorded data, and 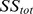 is the total sum of squares.

For the data analysed in the Results section, we assumed the parameter 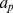 to be negligible - 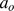 was the sole parameter fitted to the data. The single-unit place field data on the linear and circular tracks (see first two subsections in Results) has been obtained from electrodes in distal parts of area CA1 of the Hippocampus (closest to the subiculum), which receive few connections from the neural path integrator (MEC), as opposed to proximal CA1 [41]. These recorded place cells were probably mainly driven by sensory information (subiculum, LEC) instead of path integration information (MEC) [41], [42], which is why we assumed 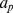, the parameter accounting for path integration accuracy, to be negligible for these specific datasets.

Since the simplifying assumptions made by the model presented here are too strong for real-world environments, and since place cell firing is influenced by many more factors other than environmental geometry, such a simple model cannot yield highly accurate predictions of electrophysiological recordings. However, if place cells integrate information in a Bayesian fashion, and if the sizes of their place fields are correlated with uncertainty, then even this simple model should be able to approximately account for the distribution of place field sizes and their dependence on the distances to boundaries and landmarks in the environment. For example, place fields should be smaller close to boundaries and larger far from boundaries. In the Results section, we will compare these predictions of the Bayesian model to data recorded from rat place cells in different environments.

Equation (7) can be extended to only include subsets of observed objects (see Discussion) by introducing a set of binary variables 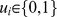 indicating whether a certain object observation is being used in the uncertainty estimation. If 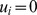, then the probability of observation 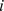 does not influence the posterior probability. Thus, in the one-dimensional case, the observation probabilities will be
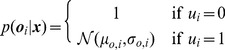
(8)


If we insert equation (8) into equation (4) calculating the mean and uncertainty (standard deviation) of the ‘best guess’ location, and solve for the standard deviation (see [40]), we get the following extended expression representing the uncertainty depending on the distances of a subset of the observations:
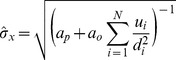
(9)where 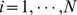 indexes one of 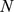 objects, boundaries, or obstacles. 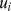 can be fitted using e.g. a non-linear optimizer or a brute-force approach - trying all possibilities - if the number of obstacles is small enough (in the Results section, we have adopted the latter approach).

### Bayesian inference on the neuronal level: a possible model

The hypotheses outlined in the Introduction section imply that, physiologically, the firing fields of place cells should shift and shrink in a statistically optimal fashion. This might be caused by a large number of possible mechanisms (see e.g. [43] for some proposed implementations of Bayesian inference in brains, and the Discussion section). We have chosen to implement a different solution for Bayesian inference in spiking neuronal networks, based on coincidence detection. We report simulation results of this neuronal Bayesian inference model in the last subsection of Results. This neuronal model rests on the following assumptions:

#### Inference using coincidence detection

A mechanism for obtaining a Bayesian posterior requires a multiplication of probability distributions. In a network of spiking neurons, such multiplication might be implemented by coincidence detection [44], a mechanism that hippocampal CA1 neurons have been observed to exhibit [45]–[47]. This particular implementation of multiplication is a hypothesis that our proposed conclusion does not depend on, since multiplication could also be implemented neuronally in several other ways (e.g. [48]). However, we chose this one for its simplicity and computational efficiency. Furthermore, a number of neuronal network models capable of performing Bayesian inference have been proposed before [43], [49]–[52]; nevertheless, none of these methods are fully compatible with the anatomical properties of the HEC and the physiological evidence from place cells (see Discussion). For this reason we chose to implement a novel solution for Bayesian inference in spiking neuronal networks, based on coincidence detection, and inspired by sampling-based approaches to represent probability distributions [53]–[55].

#### The temporal resolution of coincidence detection is in the right range to approximate multiplication

Bayesian inference requires multiplication. Multiplication by coincidence detection only works well within a certain range of temporal resolution of the coincidence detection. If the temporal resolution is too high, very few inputs, or even one input, can elicit output spikes, in effect leading to an addition of the inputs instead of a multiplication. Too low a temporal resolution on the other hand could lead to very sparse output spikes, leading to a displacement of the output firing field and destroying the statistical near-optimality (or, in the extreme case, to zero output spikes). The coincidence detection properties of noisy integrate-and-fire neurons have been analysed in two studies [56], [57] (although their analyses are based on a simple spiking neuron model, recordings by [56] indicate that these expressions closely model the coincidence detection behaviour of biological neurons in vitro). According to [57], the temporal resolution can be approximated based on the standard deviation of the fluctuation of the membrane potential 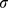, the membrane time constant 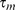 and the amplitude 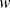 of the postsynaptic potentials (PSPs) as follows:
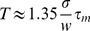
(10)


Inserting standard values observed in vivo in area CA1 of the Hippocampus into equation (10) (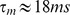 [58], [59], 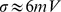 [60], and 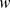 just under the 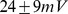 necessary to discharge a place cell [61]) yields around 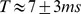. The temporal resolution of the coincidence detection in hippocampal CA1 neurons has also been measured in vitro, and is of the same order of magnitude. For example, Jarsky et al. have found that CA1 neuron firing upon perforant path input spikes is strongly facilitated by synchronous spikes from Schaffer-collateral (SC) synapses arriving within 5–10 ms, but is otherwise unreliable if no synchronous SC input is present [45].

This temporal resolution constant 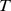 is small enough to approximate multiplication (see Results), but sufficiently large to allow enough coincidences to form a place field. Even with very sparse information, e.g. in rat experiments under total darkness [62], [63] in which the place fields presumably arise mostly from grid cell input, place cells might receive up to 200–20,000 incoming spikes per second (based on around 100–1,000 connections between grid cells and a place cell [4], [64], [65], and a grid cell firing rate around 2–20 Hz [16], [66]). Given the temporal resolution of 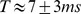, this spike rate is sufficient to elicit the empirically observed CA1 place cell firing rates of around 1–10 Hz (e.g. [42], [67]) in locations where many grid cells firing fields overlap.

#### Approximating a Bayes-optimal location estimate

Place cells should approximate a Bayesian posterior according to hypothesis 2, as expressed in equations (1) and (2). Neuronally, each border cell could represent a boundary proximity probability distribution 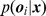, if we assume that firing rate distributions are correlated with probability distributions (cf. hypothesis 1). The MEC grid cell path integrator could provide the prior location distribution 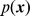. Although a single grid cell cannot provide an unambiguous estimate, having many firing fields, an ensemble of multiple thresholded grid cell inputs yields a single firing field (or few firing fields) in small environments, as pointed out by grid cell-driven place field models [3], [4]. This reduction to one or few firing fields works both with additive inputs, as in most rate-coded neural network models, and with multiplications of inputs.

**Figure 4:**
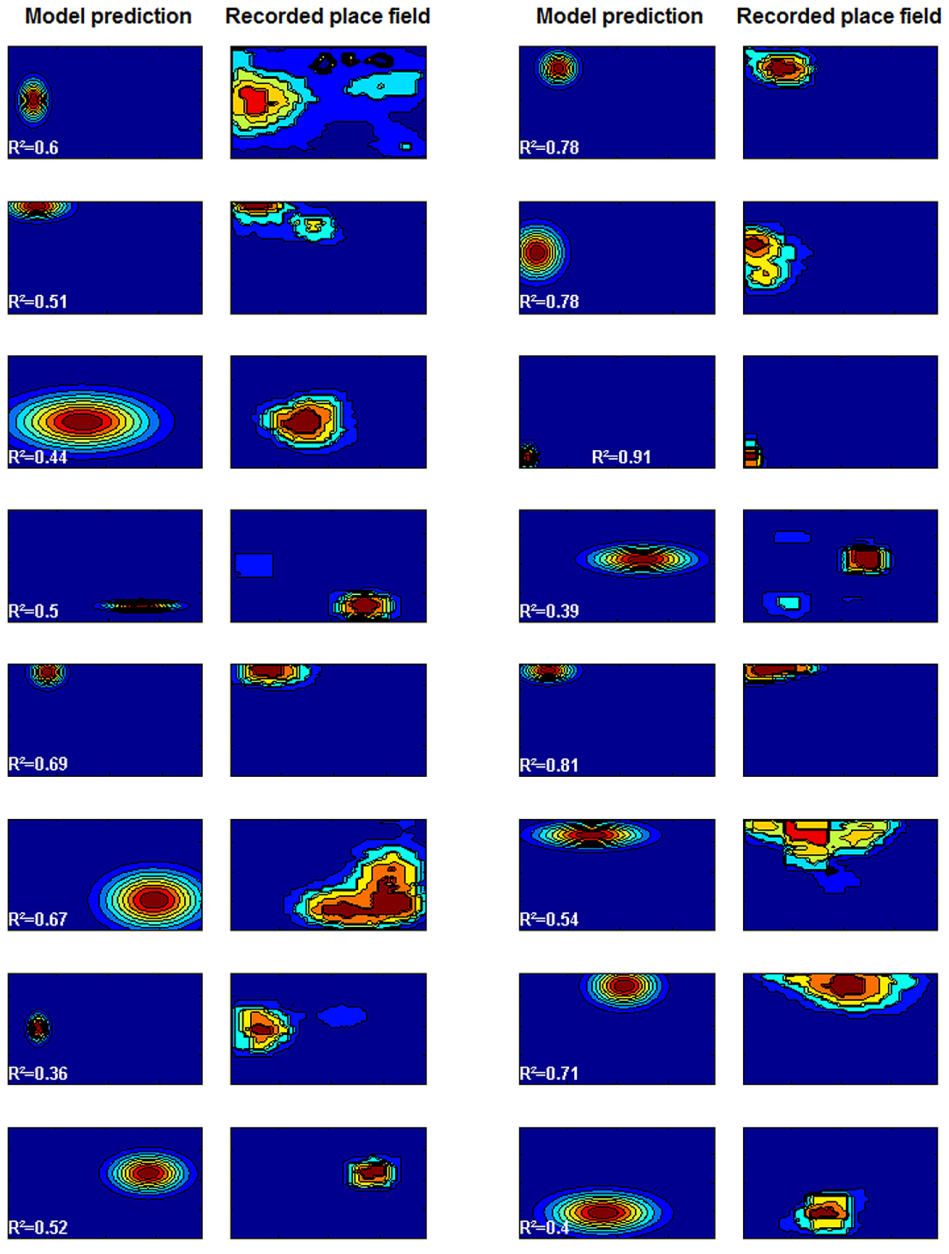
Neuronal implementation of Bayesian inference based on coincidence detection. This simple integrate-and-fire model contains only three spiking neurons, and shows their spikes over 5 simulated seconds. Each plot shows the spikes (blue dots in bottom rows), as well as the corresponding instantaneous firing rate or spike density. First row: a simulated grid cell (pre-defined firing rate function), used as the prior. Second row: simulated border cell (pre-defined firing rate function), used as the observation likelihood. Third row: simulated place cell, representing the posterior, firing only when all incoming inputs are coincident (i.e. they occur within a small time window). The Gaussian drawn over the mean and standard deviation of the noise-filtered spikes represents the place field, and approximates the Bayesian optimum. Bottom row: plot of the membrane potential of the place cell.

Integrate-and-fire spiking neurons are able to approximate the multiplication of their inputs by making use of coincidence detection (see Figure 4). Thus, such neurons can represent a posterior (i.e. a product of probability distributions). If we represent the spike train of each neuron using a function 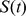, which at a given time 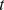 is 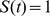 if the neuron has fired a spike within the time interval 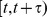, and 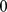 otherwise (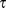 being the time discretization parameter of the model, which we set to the temporal resolution of coincidence detection in place cells - see Text S2 in the Supporting Information), then the spike train of the place cell computing the posterior, 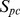 can be expressed using the spike trains of its 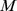 input neurons, 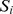:
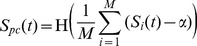
(11)


Where 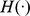 is the Heaviside step function, and 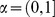 is the proportion of input neurons required to spike within 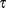 time in order to elicit an output spike in the place cell. See Text S2 in the Supporting Information for the derivation, and for arguments why this expression approximates multiplication. Using equation (11), we can express the probability 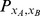 that the rat is on a path between the locations 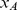 and 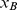 during 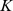 time intervals of duration 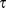 (represented by 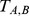), using the spike train of a place cell presumably representing the outcome of the Bayesian inference process 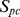, the spike trains representing of 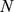 grid cells 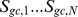, and the spike trains of 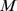 border cells 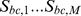:
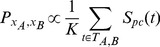
(12)

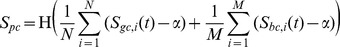
(13)


Equations (12) and (13) describe how Bayesian inference can be implemented in a spiking neuronal network, approximating the posterior probability distributions with spikes of the place cell which are viewed as samples of that distribution (see Text S2 in the Supporting Information for the derivation, and for a formulation of coincidence detection as rejection sampling; and see Figure 4 for simulation results using integrate-and-fire spiking neurons).

## Results

### Place field sizes on a linear track

Figure 1 shows this prediction of the Bayesian model in a rectangular environment, and compares it to single-unit recordings of the place cells in area CA1 of the hippocampus of ten male Lister Hooded rats (data from [68]). The rats ran on a narrow rectangular track with food cups at both ends. These sizes were also used to generate the model predictions. In the following, x denotes the distance of the rat from the eastern boundary, 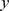 the distance from the southern boundary, and 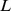 and 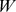 the constant length and width of the environment (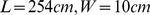 [68]). The model was instantiated with the four boundaries of the environment, and the uncertainty at each point of the track calculated by multiplying the separately calculated 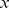 and 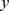 uncertainties 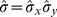, which are assumed to be independent on this track (see Methods).
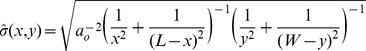
(14)


The y-axis of Figure 1 shows the total place field area of the recorded place cells, in bins of 1.9 cm. Under the hypothesis that uncertainty is correlated with place field size (H2), equation (14) implies that the biggest place fields should be in the center of the track. Since both the distance from the east boundary and from the north boundary influence the uncertainty, it also implies that at each position along the length of the track, there should be multiple uncertainties, depending on whether the rat is close or far from the side borders (the south/north border), which is shown by the two red lines in Figure 1 (the thin red line corresponds to the rat running close to the south/north border, and the thick red line to it running in the center, far from those borders). The parameter 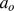 in equation (14) was adjusted using a coordinate descent algorithm. Using this single parameter, the model can explain why place fields were bigger when closer to the center of the track. Most of the recorded place field sizes (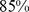) fall between the boundaries of the model.

### Place field sizes on a circular track with objects

Figure 2 shows the results of the model in a more complex environment, comparing the sizes of place fields of recorded place cells of two male Fischer-344 rats in an experiment performed by Burke et al. [42], in which the rats were running on a circular track with 106.7 cm diameter and 15 cm width. The track contained a barrier with food trays on each side to motivate the rats to run along the track, alternating between clockwise and counter-clockwise laps. It also contained 8 randomly distributed objects, and was otherwise featureless. The Bayesian model, equation (7), was fitted to the recorded data, using 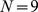 observations (the 8 objects, and the barrier). Uncertainty was calculated in one spatial dimension, which corresponds to the distance of the rat from the barrier along the track.

The single-parameter model achieved correlations of 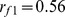 for rat 1 and 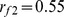 for rat 2 between the smoothed place field sizes and the fitted model - see Figure 2 (the probabilities of getting correlations as large as these values by random chance are negligible: 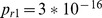 for rat 1 and 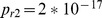 for rat 2). The average place field sizes clearly have a non-random structure, with the minima corresponding to the locations of the 8 objects and the barrier, as predicted by the Bayesian model (the null hypothesis of the data being random can be rejected with high confidence, with 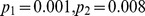 for the two rats according to a chi-square goodness-of-fit test of the place field size data against a normal distribution).

On the other hand, it is plausible that the residual errors, i.e. the model subtracted from the average place field sizes, are randomly drawn from a normal distribution, implying that the model explains a significant part of the non-random structure (the null hypothesis of the errors being random cannot be rejected according to a chi-square goodness-of-fit test of the residual errors against a normal distribution, with 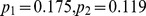 for the two rats). Some recorded place cells had multiple place fields [42], in which case the predicted uncertainty was calculated for each place field separately.

**Figure 5:**
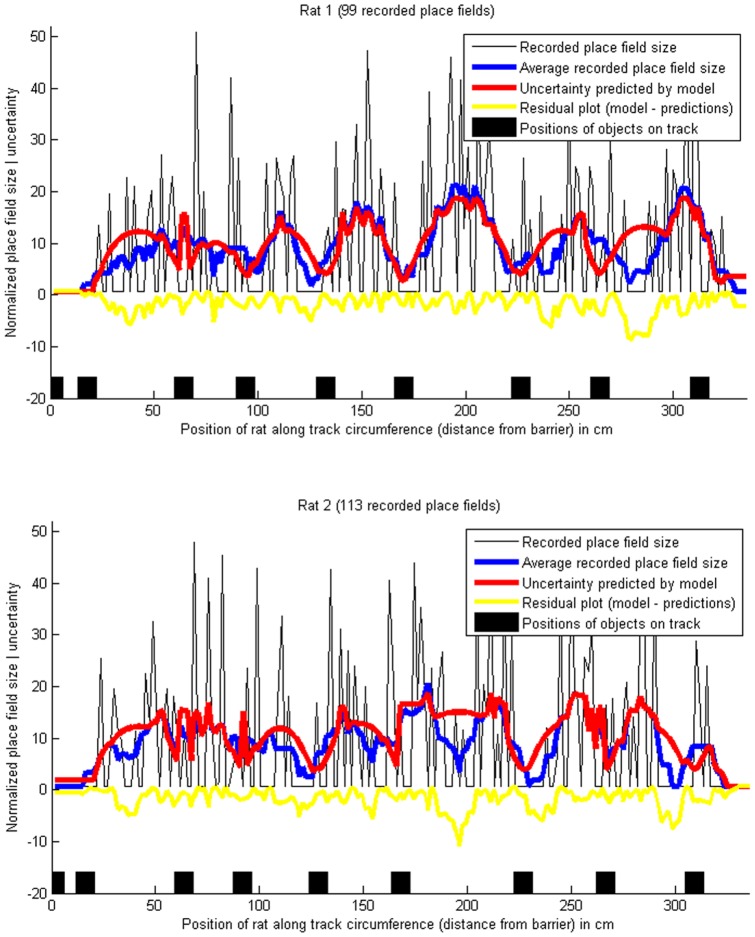
Density of place cell spikes, and predicted uncertainty, on a circular track with objects. The blue lines show the smoothed (averaged) density of place field spikes, i.e. the number of spikes across all recorded place cells for each centimetre of the track, normalized to a mean of 0 and variance of 1. The red lines have been obtained by summing Gaussian distributions, one for each place cell, with the means set to the center of each place field, and the standard deviations set to the location uncertainties (hypothesized to be correlated with place field sizes, see H2) as above. The exact amplitude of the spike density at each location depends on the place cells firing rate, which is influenced by many non-spatial factors such as running speed [67], but the shape of the curves is comparable. Pearson's correlation coefficient between the recorded place field sizes and the predicted uncertainty was 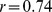 for rat 1 and 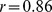 for rat 2. The proportions of explained variance were 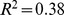 for rat 1 and 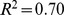 for rat 2. (Data from [42]).

In Figure 2, the x-axis shows the positions of the means (centroid) of the recorded spikes of each place field, and the y-axis shows the size of the fields, derived by calculating the standard deviations of the spike positions. This makes these place field sizes directly comparable to the uncertainties calculated by equation (7), provided that the place fields resemble Gaussians, being approximately symmetric, and having the highest spike density around the mean (centroid). If this was not the case - if the recorded place fields were not approximately Gaussian -, the spike densities would deviate from the prediction of the model. Figure 5 compares the spike densities of all recorded spikes to the densities predicted by the model, achieving correlations of 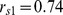 for rat 1 and 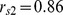 for rat 2 (the probabilities of getting correlations as large as these values by random chance are negligible: 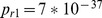 for rat 1 and 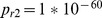 for rat 2).

### Place field sizes after changes in the environment size

Changes in the environment have been shown to influence place fields. In order to show that the Bayesian model does not violate the observed effects, and can predict place field size in novel environments, we have applied it to the data presented in [7] for evaluating the BVC model, and originally reported in [69]. The data was recorded from six rats foraging for food in four different environments: a small square of size 61×61 cm (environment A), a large square of 122×122 cm (environment B), and a horizontal and vertical rectangle of 61×122 cm and 122×61 cm (environments C and D). 12 of the 28 recorded place fields were discarded from the dataset because they were asymmetric and did not fit a Gaussian distribution (see Discussion for possible model extensions). For the remaining 16 place fields, the parameters of the model were adjusted using the data from two of the four environments, C and D. The means and standard deviations of the Gaussians used to represent the place field in the 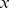 and 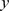 dimensions were obtained by using a least squares fitting procedure, and the parameter 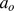 calculated from the known distances and standard deviations using equation (7). This equation also allowed calculating the predicted place field size, i.e. the standard deviation of the representing Gaussian, in the remaining two environments, by using appropriately scaled distance relations.

Then, the predicted and recorded place fields were compared at each point in the environment (see Figure 3). Figure 3 shows the results for environment B (the large square environments), achieving a mean proportion of explained variance of 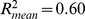. This fit of the model predictions was compared against the optimal fit possibly achievable by Gaussian functions, calculated by fitting Gaussians to each actual firing field in the B environments using a least square errors procedure. This optimal fitting procedure yielded 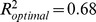 on average, which is not statistically different from the model fit (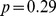 on a paired t-test over all individual place field 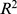 values). This shows that the Bayesian model can make predictions which fit the data almost as well as optimally fitted Gaussian functions. The difference between the fit of the model and of this optimal fit is statistically insignificant.

### Place field sizes from subsets of observed objects

The model used so far makes a number of simplifying assumptions, which yield a very simple mathematical form with up to two parameters - see equation (7) - and already provides reasonable predictions of experimental data (see above). However, the accuracy of the model can be improved by relaxing some of these assumptions, at the expense of simplicity (see the Discussion section).

One way to improve the model accuracy is to allow place cells to be driven not by every single boundary and obstacle in the immediate environment, but only by a subset of these objects. Equation (9) allows the calculation of uncertainties taking into account a subset of observations (see Methods). This extension introduces 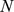 additional model parameters for the 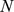 binary variables 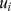 specifying whether or not observation 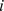 is being taken into account.

**Figure 6:**
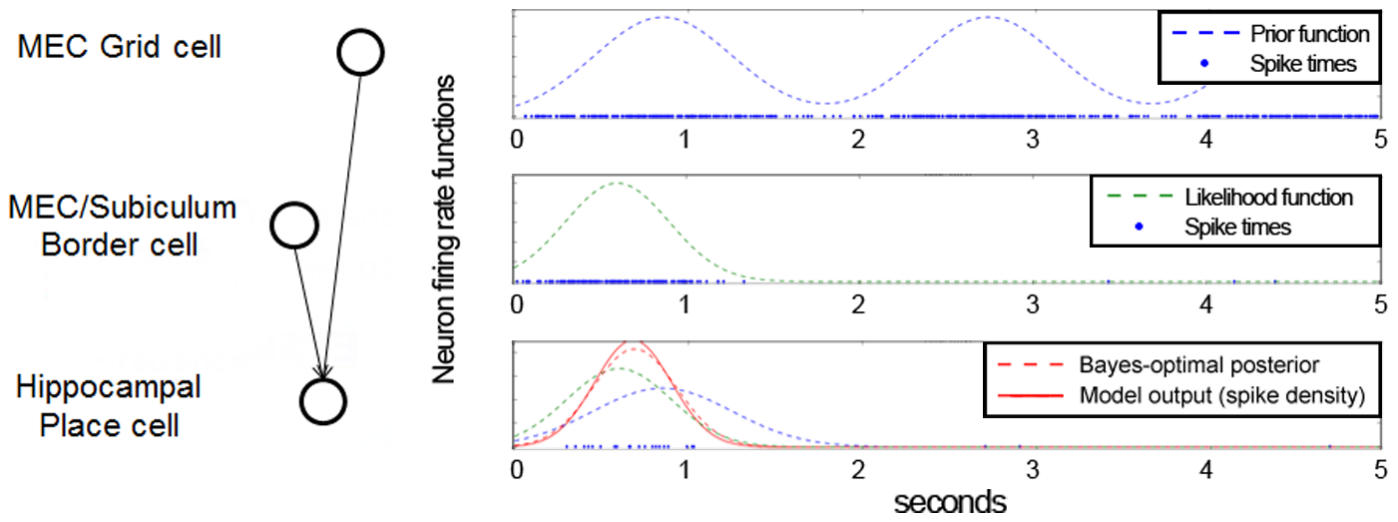
Place field sizes, and predicted uncertainty, on a circular track with objects, using the extended model. The blue lines show the smoothed place field sizes (10-point moving average), normalized to a mean of 0 and variance of 1, and the red lines show the location uncertainty predicted by the extended Bayesian model (which takes into account only a subset of the objects on the track at each point). Pearson's correlation coefficient between the recorded place field sizes and the predicted uncertainty was 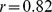 both for rat 1 and rat 2. The proportions of explained variance were 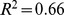 for rat 1 and 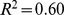 for rat 2. (Data from [42]).

Fitting this extended model to the data recorded on the circular track with objects, significantly increases the model fit - instead of explained proportions of variance 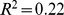 for rat 1 and 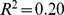 for rat 2, the extended model achieves 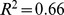 for rat 1 and 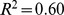 for rat 2 (see Figure 6). However, this extended model uses 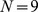 more parameters than the original model (8 objects on the track, plus the barrier with the adjacent food trays). To take into account the number of parameters relative to the number of data points, we also compare the adjusted 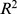 values (which we denote by 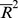). Instead of 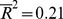 for rat 1 and 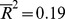 for rat 2, the extended model yields 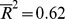 for rat 1 and 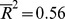 for rat 2 after adjustment by the number of parameters.

Further possible extensions of the model, such as allowing skewed place fields, will be described in the Discussion section.

### Bayesian inference on the neuronal level: a possible model

As argued above, the sizes of place fields should be dependent on incoming sensory information, in order to approximate the statistically optimal location of the animal, and the uncertainty associated with it. Mathematically, this means calculating a Bayesian posterior (see Methods). We have already presented some evidence that place cells might be able to approximate such Bayesian calculations in the previous sections. Here we extend this idea by suggesting a tentative model of how these calculations might be implemented on the neuronal level.

A spiking neuronal network could implement the multiplication operation required for calculating a Bayesian posterior by making use of coincidence detection. Figure 4 shows a simple example of a place cell receiving input from only one grid cell (path integration) and one border cell (observation). The place cell is modeled using a current-based integrate-and-fire neuron model [70] (membrane time constant 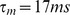, synaptic time constant 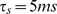, resting potential 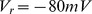, spike threshold 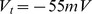, synaptic weights 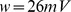). Synaptic inputs are modeled as spike trains drawn from non-homogeneous Poisson processes, with firing rates controlled by Gaussian distributions (see dashed lines in the figure) to approximate the symmetric firing fields of grid cells and some border cells. The Brian simulator was used to simulate the place cell and to plot Figure 4 [71].

In the figure, the place cell only fires when both the grid cell and the border cell inputs arrive within a small time window. This leads to a shifting of the place field - the place cell combines both types of information, and forms the place field at a location specified by the weighted average of the grid field and border field location, the weighting depending on the uncertainties (field sizes) of the inputs. Thus, the place field is located between the grid and the border field, but closer to the border field because it is narrower (more accurate).

**Figure 7:**
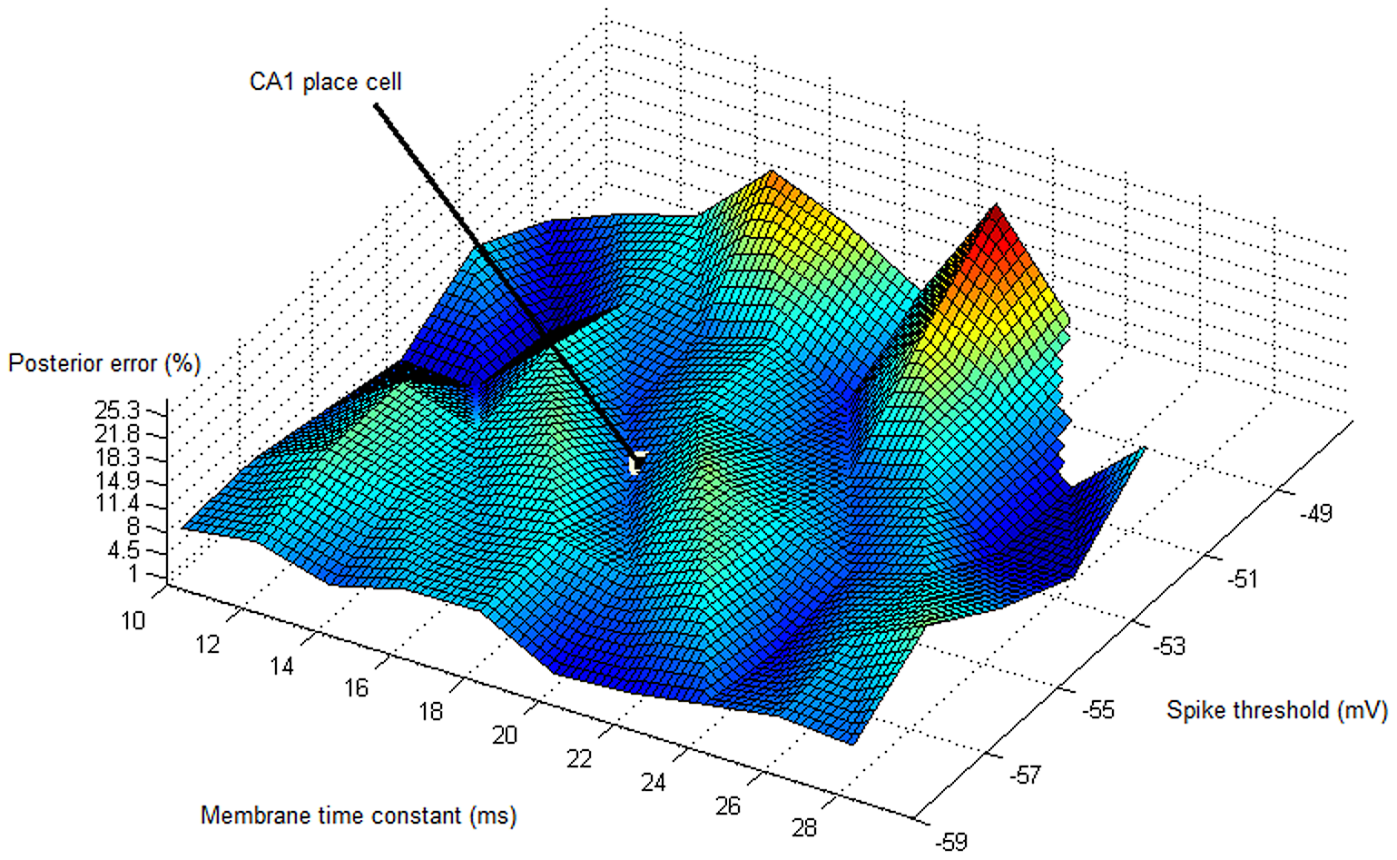
Errors of coincidence-based multiplication based on a simple integrate-and-fire model. The altitude shows the error (lowest point: 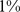, highest point: 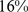, error at CA1 place cell parameters: 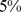), and the 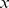 and 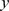 axes show the dependence of the error on the membrane time constant 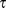 and the spike threshold 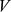 respectively. Interestingly, the parameters of some CA1 place cells (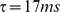, 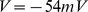) fall into one of the local minima of error; and no hippocampal place cell reaches the area of maximum error.

Figure 4 is intended to illustrate the concept of inference by coincidence detection. The model relies on the fact that if the threshold of the output neuron is set high enough to only allow output spikes on synchronous input spikes, then the output neuron performs approximate multiplication, as required by Bayesian inference. The approximation error mainly depends on two parameters of the output neuron: its membrane time constant, and its spike threshold voltage. For our purposes, we define the approximation error as the absolute difference between the posterior mean estimated by the model, and the mean of the exact posterior according to Bayes' rule (the error of the posterior mean is most relevant for a location model, since the statistically optimal location estimate is located at the mean of the posterior distribution under Gaussian assumptions). Figure 7 shows how this approximation error depends on these two parameters. For an analytical discussion of the coincidence detection properties of integrate-and-fire neurons, see [56], [57].

## Discussion

We have attempted to highlight the usefulness of Bayesian models in explaining information combination in place cells. Although such models are too simple to explain all firing properties, their predictions fit the data quite well given their simplicity (low numbers of parameters), which is an important property of good models [72]–[74]. We have compared such model predictions to three different datasets recorded from rat place cells in different environments in the Results section, using firing field size as a measure of uncertainty. Our results suggest that the ‘Bayesian brain’ hypothesis might be useful in trying to understand information processing in Hippocampal place cells, not just at a computational level as has been suggested many times before [8]–[11], but also at the neuronal level.

Bayesian spatial cue integration has been investigated before on the behavioural level. Nardini et al. [75] investigated cue integration in human children and adults, using a paradigm in which subjects had to return an object to its original place, either given only landmark information, only self-motion information, or both. Their results suggest that adults are able to reduce the variance (uncertainty) in their response by integrating different spatial cues in a statistically near-optimal fashion. Cheng et al. [11] reviewed animal experiments, arguing that the integration of different spatial cues might be partially explained by Bayes' rule - for example, pigeons seem to assign weights to information from different landmarks using Bayesian principles. Therefore, in contrast to previous work, this paper significantly extends these ideas by directly comparing the predictions of Bayesian spatial cue integration to physiological data recorded from rat place cells, and argues for the plausibility of this cue integration mechanism on the neuronal level.

The claim that perception (spatial or otherwise) is based on Bayesian inference, implemented physically as a neuronal mechanism, has been criticized for multiple reasons [15]: the lack of strong physiological evidence in favour of the Bayesian hypothesis (most existing evidence to date is behavioural, coming from ‘Bayesian psychophysics’ [15], [76]), the arbitrary choice of prior functions in favour of simplicity in many of these models (instead of the choice being based on empirical data), and the ability to explain Bayes-optimal perception in cue integration in some paradigms *without* a Bayesian mechanism, by implementing reinforcement learning.

In this paper we have argued that firing field properties of single place cells resemble the outcomes of Bayesian inference processes. Following the advice of [77] we have generated quantitative experimentally testable predictions, and compared them with empirical results. Thus, in contrast with the view that *‘Bayesian models do not provide mechanistic explanations currently, instead they are predictive instruments’* [15], we provide one of a few existing pieces of empirical evidence in favour of the idea that the brain might represent uncertainty at a neuronal level, and that there are some neuronal level mechanisms approximately conforming to Bayesian principles. Our results therefore contribute to the *‘current challenge for these* [Bayesian] *models* [is] *to yield good, clear, and testable predictions at the neural level, a goal that has yet to be satisfactorily reached’* [15].

### Bayesian localization

Bayesian cue integration might also play a role in the more complex problem of maintaining a near-optimal location estimate through time, despite noise and accumulating errors. In robotics, one popular family of solutions for maintaining statistically optimal location estimates is called Bayesian localization (an example algorithm from this family would be the Kalman filter) [38]. Given some simplifying assumptions, Bayesian localization can be performed by the following three computations at each time step, in order to maintain a statistically optimal, error corrected location estimate:

1. **Path integration.** Updates the prior location belief with (possibly erroneous) movement vectors using a motion model at each time step.
2. **Correction.** A Bayesian inference mechanism that corrects the location belief using observations.
3. **Update.** Finally, the path integrator's estimate is updated to the corrected estimate.

There is ample evidence in literature that the HEC is able to perform step 1 [17] - grid cells update their firing with each movement. We have presented evidence in the Results section for step 2, strongly suggesting that place cells might be able to perform approximate Bayesian computation. With respect to step 3, there is anatomical evidence that such an update could happen - place cells can project back to grid cells and influence their firing [78]–[80]. Such back-projections might serve the role of providing environmental stability for the grids [81], and prevent the accumulation of error during path integration [3], [18], [82]. They are also postulated in a model of grid-cell based error correction, which shows how the redundant modular coding in the entorhinal cortex might constitute an exponentially strong population code - it can *‘produce exponentially small error at asymptotically finite information rates’* [83] (however, this model does not account for location correction using observations). The idea of back-projections from grid cells to place cells is supported by recording evidence showing that grid cell representations become erroneous, less gridlike, and expand in field size in novel environments [66]; and recent evidence indicating that deactivating the hippocampus extinguishes grid fields [84].

Thus, the Hippocampal-Entorhinal Complex might be able to implement Bayesian localization and maintain approximately statistically optimal location estimates through time, despite accumulating errors. Entorhinal grid cells are able to integrate movement signals [17]. Bayesian cue integration in place cells (see Results section) might be the mechanism performing the correction step and then, after near-optimal cue integration, the corrected location estimate would update grid cells (the neuronal path integrator) through the place cell back-projections.

Phase resetting presents a plausible mechanism by which to perform this update step. It has previously been suggested that error correction in oscillatory interference models of grid cells might be implemented through phase reset, the resetting of the phase of intrinsic oscillations in MEC grid cells [81], [85], [86]. Therefore, when entering a new environment, connections might form between place cells and grid cells firing simultaneously (i.e. between cells with coinciding firing fields), to anchor the grid field representation to environmental features such as boundaries. These connections could induce a reset of the intrinsic oscillation phase of the grid cell when the grid field shifts (e.g. due to path integration errors) [85]. The changed oscillation phase would lead to a displacement of the grid field back to the center of the place field, because grid cell firing fields arise from the oscillatory interference patterns between background theta oscillations and the intrinsic oscillations in the grid cell in oscillatory interference models, with the grid cell firing rate being highest when the phases coincide [87].

There is some recording evidence showing that single incoming spikes can indeed reset intrinsic oscillation phases in cells of the entorhinal cortex [88]–[90]. Because a single postsynaptic potential suffices, the probability of phase reset occurring depends on the firing rate(s) of the place cell(s) connected through the back-projections. Thus, as the animal is running through the place field, the firing rate within the grid field might gradually adapt to the firing rate of the place field, and the fields would become aligned, completing the update step.

### Possible extensions

There are some properties of place fields which the model presented here, in its simplest form, while not inconsistent with, cannot account for. The basic uncertainty estimation, equation (7), does not account for place cells driven by only a subset of the objects in the environment, instead of all of them, however, some place fields have been observed to be controlled by specific landmarks [91]. Equation (9) makes it possible to parametrize which subset of the object distances are taken into account for the uncertainty calculation, yielding a significantly better model-data fit on the track with multiple objects (see Results).

Although the equations used in the Results section use a single Gaussian distribution to model a place field, this model can be used to model place cells with multiple place fields in a straightforward fashion, by calculating a separate uncertainty value for each place field using the respective distances of objects from the place field centroids. Thus, multiple uncertainty values can be associated with each place cell, one for each place field - as in Figure 2 for example, in which many of the plotted place fields belong to multi-field place cells (see [42] for the distribution of single-field and multi-field place cells in this dataset).

Further phenomena not explained by the simple model include asymmetric place fields that are frequently found in area CA1 of the hippocampus, and the observation that place field sizes seem to increase along the dorso-ventral axis of the hippocampus [67]. Asymmetric place fields could potentially be modelled using skewed probability distributions such as the Skew-Normal Distribution [92] as observation likelihoods instead of Gaussians, using a similar approach to the one described in the Methods section. The grid cell input to a place cell is usually symmetric, but the firing fields of border-related cells can be skewed [22], [23], which might give rise to asymmetric place fields. The skewness parameter of an asymmetric probability distribution (such as the Skew-Normal Distribution) in such an extended model might increase as a function of familiarity with the environment (time spent in the same environment), in order to model the experience-dependent asymmetry of some CA1 place fields [93]. The mean and variance of such a distribution could be estimated similarly to the approach proposed in the Methods section. Future work, and experimental data from place cells recorded over extended periods of time, will be needed to verify how well such an asymmetric model could account for skewed place fields.

It is interesting to note, with respect to the fact that the place field sizes increase along the dorso-ventral hippocampal axis, that the same field size increase has been observed in grid cells in the medial entorhinal cortex [94]. Since grid cells are hypothesized to play a role in driving place cell firing, both in our model and in previous models [3], [4], this might account for the place field size gradient. In an extended model taking into account the spatial configuration of the hippocampal-entorhinal complex, if the dorsal grid cells are adjusted to have small firing fields and the ventral ones large firing fields (50 cm–3 m, see [94]), this will lead to a similar gradient in the resultant place fields, given that the grid cells at least partially drive the firing of the place cells. The role played by boundary-related inputs would mean that not every place field would fit this dorso-ventral size gradient, but on average a field size gradient could be observed in such a model.

### Related work

The Boundary Vector Cell model [24] of place cell firing also explains place fields in terms of geometric relations to environment features, although it does not suggest statistical near-optimality and does not make use of Bayesian cue integration. The objective fit of the simple model presented in this paper is not as good as the fit achieved by the Boundary Vector Cell model ([7] describes the fit of the BVC model to the data in figure 3). The BVC model could, in principle, also be fitted to the first two datasets presented in the Results section, but would require the adjustment of a higher number of parameters than there are data points and thus would not have a unique solution (Hartley et al. [7] simulated 2–4 inputs per place cell, requiring up to 7 parameters to be adjusted for each place cell; and a few additional global parameters - over 700 fitting parameters for the data in Figure 2).

The model presented here serves a different purpose; not to present a more accurate model of place fields, but rather to highlight that the information integration in place cells approximately resembles simple Bayesian computation. Our results suggest that predictions resembling in-vivo recorded place field data can be made based on a *single underlying principle: the statistically optimal combination of information*. Because of its simplicity, this model cannot fully explain experimental data, and does not achieve a fit as good as previously suggested models such as the Boundary Vector Cell model [2], [7], [24] (since it only uses a single global parameter for the results illustrated in Figures 1, 2 and 3). It has been argued that in addition to quantitative fit, simplicity and parsimony are also important and desirable characteristics for potentially valid computational models [72]–[74]. Thus, we believe it is important to consider not only models that are capable of fitting data very well, but also models that offer simple explanations, and we have described such a model, using a Bayesian framework and a single parameter.

It has been suggested earlier [29] that sensory information might be used to correct path integration error. Previous work building on this idea can be categorized into high-level models, suggesting correction mechanisms but unconcerned with the details of neuronal implementation, and neuronal-level models.

High-level models of hippocampal error correction have proposed a Bayesian information integration mechanism before [11]–[14]. Cheung et al. [14] show that featureless boundaries alone are insufficient for unambiguous localization, and propose a similar model of Bayesian localization to the one outlined here, based on the implementation of a particle filter, and replicate some experimental results on place and grid field stability using their high-level model. However, they do not account for single cell firing field data, and they do not suggest how the particle filter might be implemented in the brain. MacNeilage et al. [13] suggest Bayesian cue integration to estimate spatial orientation under uncertainty, suggesting Kalman filters (which use unimodal Gaussian probability distributions) or, alternatively, particle filters (which are capable of dealing with multimodal and non-Gaussian probability distributions) as the mechanistic implementation. Pfuhl et al. [12] also hypothesize spatial information integration to be Bayesian, choosing Kalman filters as their implementation. Finally, Cheng et al. [11] propose that spatial information is integrated in a Bayesian fashion, without suggesting a formal model or a neuronal implementation, and provide some behavioural evidence for this claim.

Kalman filters are possible to implement on biologically plausible attractor networks [51], although they have the disadvantage of being unable to deal with multimodal, non-Gaussian distributions. Taking a different approach, Samu et al. [82] have used a recurrently interconnected attractor network to correct path integration errors, using sensory information via hippocampal back-projections. Their model, like most attractor-based path integration models, relies on recurrent interconnections (which area CA1 of the hippocampus seems to lack [3]). Extending their ideas, Fox and Prescott [95] have attempted to map the hippocampal formation onto a temporal restricted Boltzmann machine (and argue that inference in their model resembles particle filtering), also modelling on a functional level but trying to adhere more closely to anatomical connectivity. However, like the previously mentioned concrete computational models, they do not model empirical data to substantiate their model. Using oscillatory interference theory instead of an attractor model as their theoretical basis, Monaco et al. [96] also use cue-driven feedback to correct location errors and to handle cue conflicts. They also reproduce partial remapping in an experiment, strengthening the mechanism the model uses to resolve cue conflicts. Cue-driven location correction is also employed in the model proposed by Sheynikhovich et al. [97], in the form of connections between view cells and grid cells, weighted using Hebbian learning.

Unlike many of these models, apart from presenting a high-level model of Bayesian cue integration, we have also attempted to suggest a tentative neuronal mechanism that might underly the implementation of approximate inference. Starting from mathematical theory, a number of implementations of Bayesian inference have already been proposed (e.g. [43], [49]–[52]), although none known to the authors in the context of HEC error correction. We believe the inference mechanism described in the Results section offers a useful contribution, because most previously published spiking neuron inference mechanisms predict anatomical and firing properties inconsistent with some empirical observations if applied to place cells. For example, the distribution population coding method [98] assumes prespecific tuning functions and a sophisticated decoding operation with unclear neuronal implementation. Inference mechanisms based on a log probability population code [52] have more plausible decoding schemes, but require recurrent connectivity and global recurrent inhibition, which have only been observed in CA3, not in CA1 place cells [99], in contrast to physiological data from CA1 suggestive of Bayesian inference (see Results section). In addition, they assume specific weight matrices for statistical optimality - which could be learned in principle, but would require a non-Hebbian learning rule. Finally, probabilistic population codes (PPC) have been widely used in modelling inference [50], recently also supported by physiological data [100]. However, PPCs have no clear way to implement learning [101], and they also require recurrent connections [50]. Furthermore, the standard PPC inference scheme assumes Poisson-like variability to allow simple addition to implement inference [43], [50], which implies a direct relationship between the absolute firing rates of neurons in a PPC and the uncertainty (standard deviation) of the encoded distribution - a relationship predicted by most inference schemes. However, it has been observed that place cell firing rates increase with the animals movement speed [67] - if place cells used a PPC with Poisson variability, or any other probabilistic encoding scheme predicting such a relationship, this would imply that the faster they would run, the more certain they would become of their location (location uncertainty would decrease with increasing running speed), which is counter-intuitive and contradicts the frequently observed trade-off between speed and accuracy [102].

The model we propose has its own shortcomings, but is simple and does not depend on specific weight matrices or variability distributions. Our aim was to show that even without additional assumptions regarding connectivity, weights, or learning, the anatomy of the Hippocampal-Entorhinal Complex might be able to implement approximate Bayesian inference. Although we were unable to substantiate this tentative model with physiological data as of yet, we hope that the reported results will encourage future research addressing the often sceptically regarded [6] mechanistic ‘Bayesian brain’.

## Supporting Information

Text S1 **Location uncertainty in the two-dimensional case.**
(PDF)

Text S2 **Coincidence detection as rejection sampling and multiplication by coincidence detection.**
(PDF)
