# Correction: Bayesian Integration of Information in Hippocampal Place Cells

**DOI:** 10.1371/journal.pone.0136128

**Published:** 2015-08-14

**Authors:** Tamas Madl, Stan Franklin, Ke Chen, Daniela Montaldi, Robert Trappl

The images for Figs 3–6 are incorrectly switched. The image that appears as Fig 3 should be Fig 5. The image that appears as Fig 4 should be Fig 3. The image that appears as Fig 5 should be Fig 6. The image that appears as Fig 6 should be Fig 4. The figure captions appear in the correct order. Please see the correct figures below.

**Fig 3:**
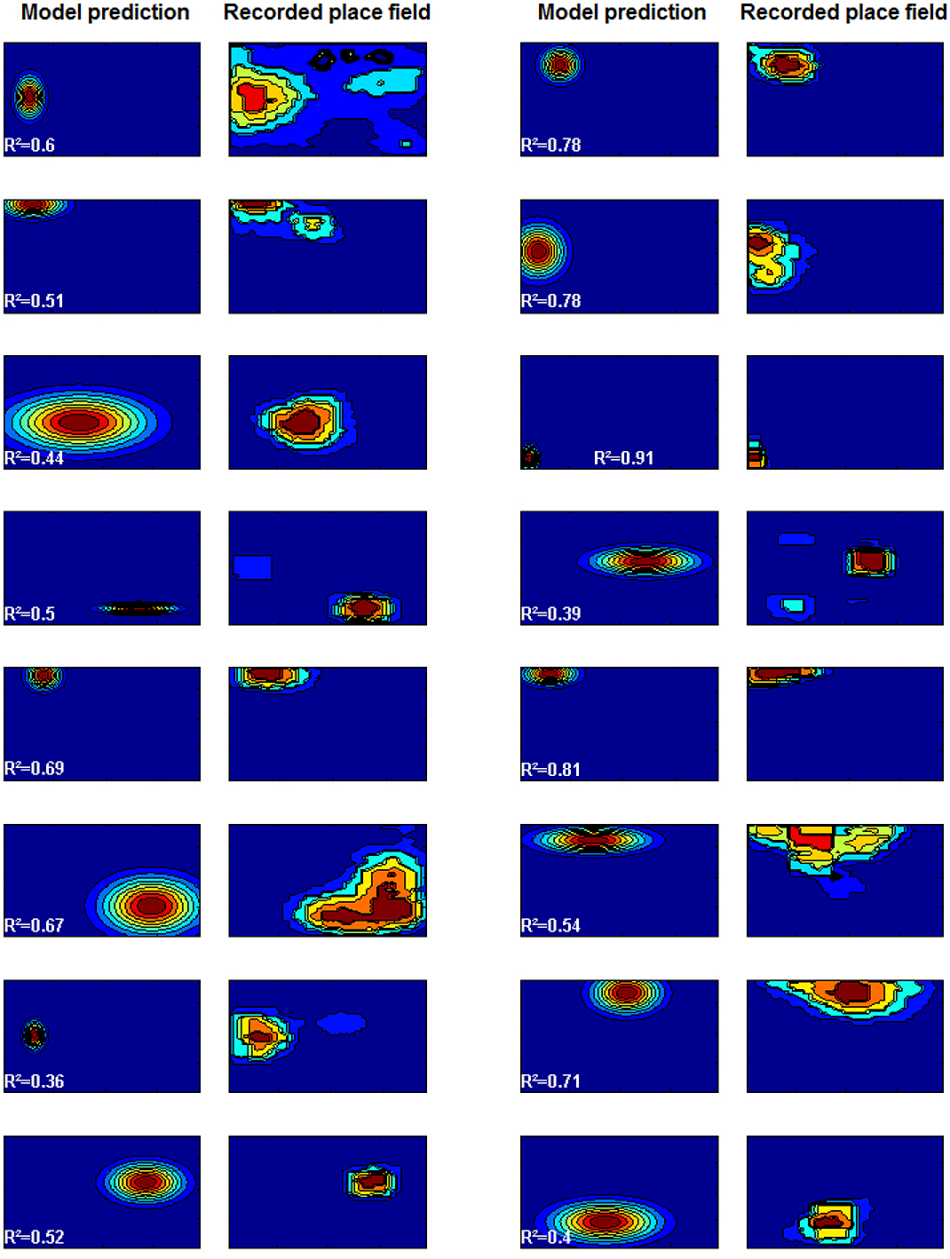
Predicted and recorded place fields in environment B. The squares represent firing rates at each point of the big square environment, with hot colors marking high firing rates, and cold colors low firing rates (the plots have been scaled to fit the page—see main text for the actual proportions of the environments). The model prediction was made based on parameters estimated from the other environments (environments A, C and D). The overall mean proportion of explained variance was *R* ^2^ = 0.60 (Data from [69]).

**Fig 4:**
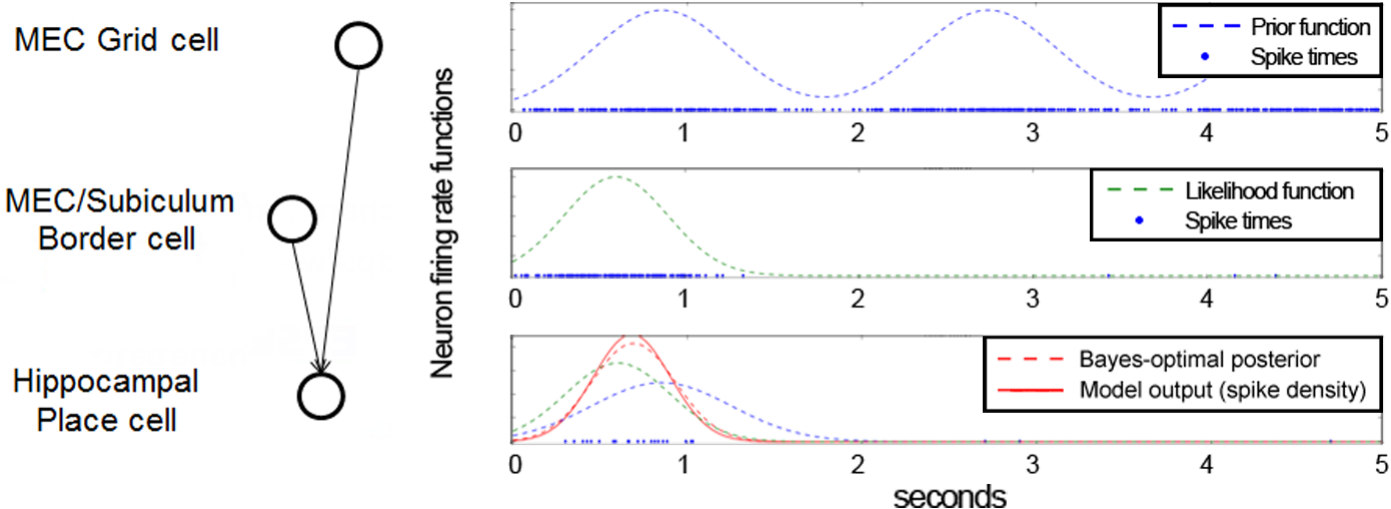
Neuronal implementation of Bayesian inference based on coincidence detection. This simple integrate-and-fire model contains only three spiking neurons, and shows their spikes over 5 simulated seconds. Each plot shows the spikes (blue dots in bottom rows), as well as the corresponding instantaneous firing rate or spike density. First row: a simulated grid cell (pre-defined firing rate function), used as the prior. Second row: simulated border cell (pre-defined firing rate function), used as the observation likelihood. Third row: simulated place cell, representing the posterior, firing only when all incoming inputs are coincident (i.e. they occur within a small time window). The Gaussian drawn over the mean and standard deviation of the noise-filtered spikes represents the place field, and approximates the Bayesian optimum. Bottom row: plot of the membrane potential of the place cell.

**Fig 5:**
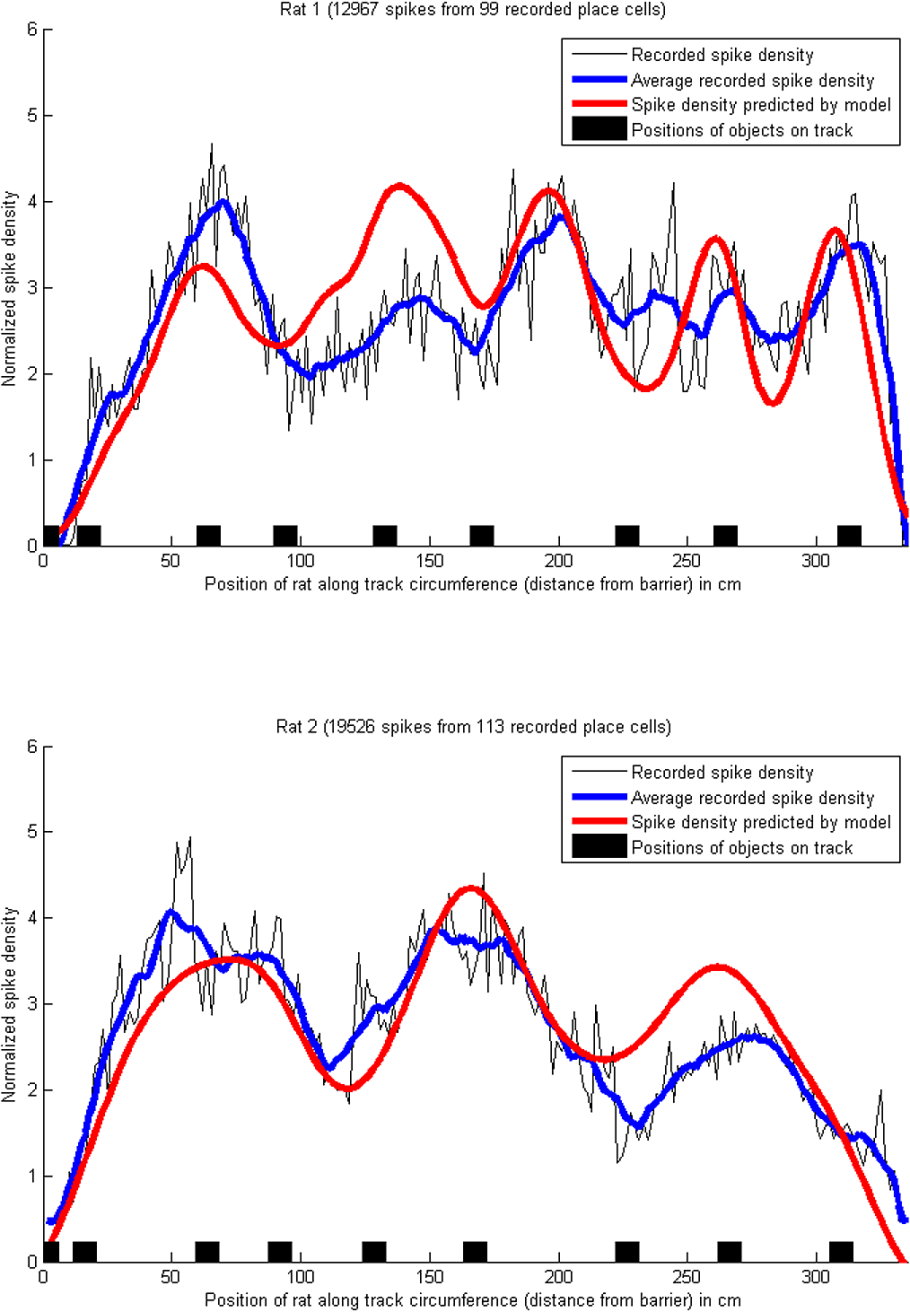
Density of place cell spikes, and predicted uncertainty, on a circular track with objects. The blue lines show the smoothed (averaged) density of place field spikes, i.e. the number of spikes across all recorded place cells for each centimetre of the track, normalized to a mean of 0 and variance of 1. The red lines have been obtained by summing Gaussian distributions, one for each place cell, with the means set to the center of each place field, and the standard deviations set to the location uncertainties (hypothesized to be correlated with place field sizes, see H2) as above. The exact amplitude of the spike density at each location depends on the place cells firing rate, which is influenced by many non-spatial factors such as running speed [67], but the shape of the curves is comparable. Pearson's correlation coefficient between the recorded place field sizes and the predicted uncertainty was *r* = 0.74 for rat 1 and *r* = 0.86 for rat 2. The proportions of explained variance were *R* ^2^ = 0.38 for rat 1 and *R* ^2^ = 0.70 for rat 2. (Data from [42]).

**Fig 6:**
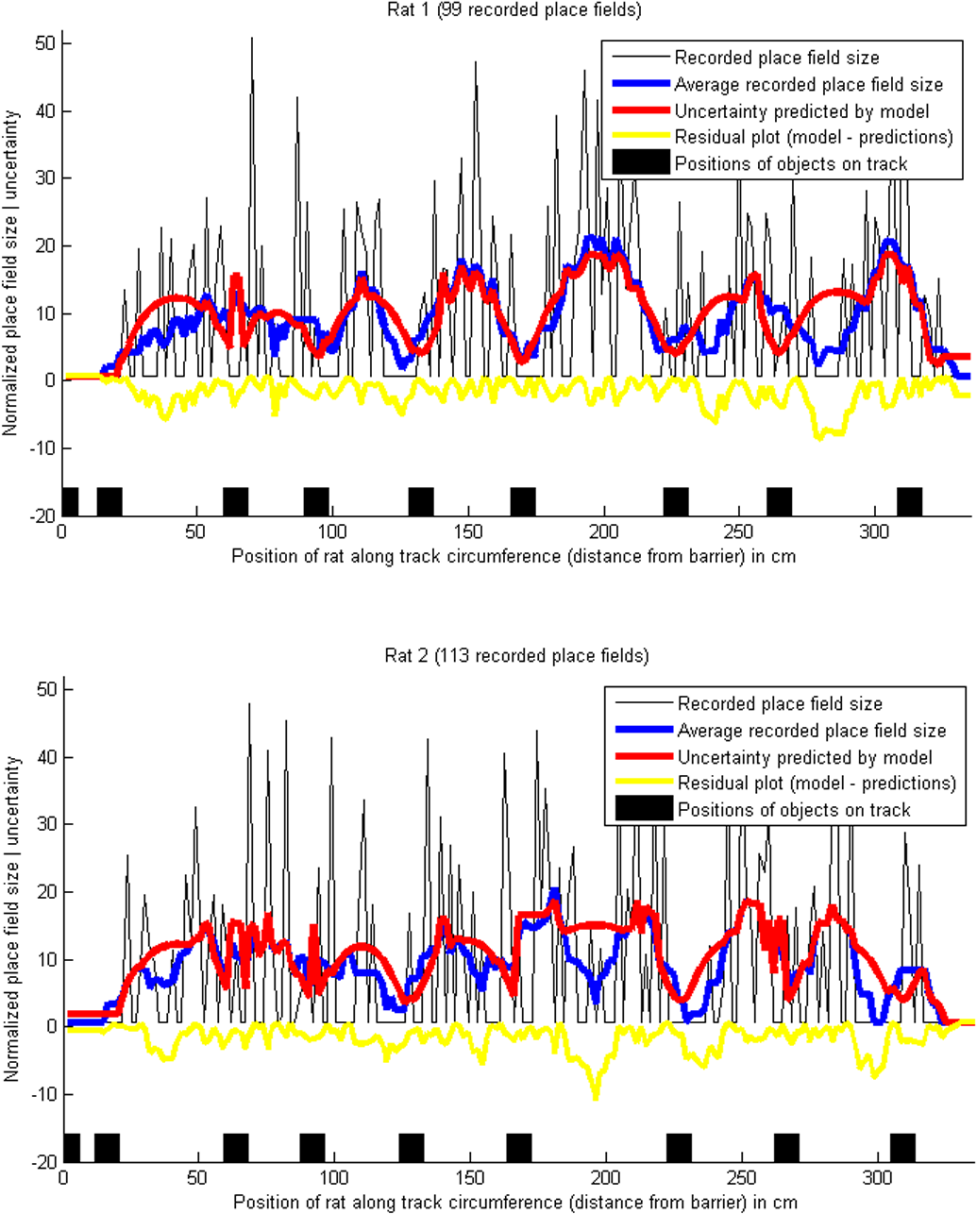
Place field sizes, and predicted uncertainty, on a circular track with objects, using the extended model. The blue lines show the smoothed place field sizes (10-point moving average), normalized to a mean of 0 and variance of 1, and the red lines show the location uncertainty predicted by the extended Bayesian model (which takes into account only a subset of the objects on the track at each point). Pearson's correlation coefficient between the recorded place field sizes and the predicted uncertainty was *r* = 0.82 both for rat 1 and rat 2. The proportions of explained variance were *R* ^2^ = 0.66 for rat 1 and *R* ^2^ = 0.60 for rat 2. (Data from [42]).
