# Supplementary material for: Bayesian Integration of Information in Hippocampal Place Cells
Source: PLoS One. 2014 Mar 6;9(3):e89762. doi: 10.1371/journal.pone.0089762 (PMC3945610; doi:10.1371/journal.pone.0089762)
Supplement: Text S1 — Location uncertainty in the two-dimensional case. (PDF) [file pone.0089762.s001.pdf]

Text S1. Location uncertainty in the two-dimensional case

As described in the Methods section (see equation (3) in the main text), under Gaussian assumptions, the probability distribution of the location given a number of observations can be calculated from

$$\mathcal{N}(\hat{\boldsymbol{\mu}}, \hat{\boldsymbol{\Sigma}}) = \gamma \mathcal{N}(\boldsymbol{\mu}_p, \boldsymbol{\Sigma}_p) \prod_{i=1}^{N} \mathcal{N}(\boldsymbol{\mu}_{o,i}, \boldsymbol{\Sigma}_{o,i}) \tag{1}$$

Where $\hat{\boldsymbol{\mu}}$ is the mean of the posterior or the 'best guess' location, $\hat{\boldsymbol{\Sigma}}$ the uncertainty (covariance) associated with this location, $\boldsymbol{\mu}_p$ and $\boldsymbol{\Sigma}_p$ are the mean and the uncertainty of the prior belief location, $\boldsymbol{\mu}_{o,i}$ and $\boldsymbol{\Sigma}_{o,i}$ are the means and uncertainties of the individual observations, and γ is a constant for normalization.

Analogously to univariate Gaussians [1], the product of a number of multivariate Gaussians is also a multivariate Gaussian. The covariance of the product in equation (1), which contains the uncertainty of the 'best guess' location, can be calculated as follows [2]:

$$\hat{\boldsymbol{\Sigma}} = (\boldsymbol{\Sigma}_p^{-1} + \sum_{i=1}^{N} \boldsymbol{\Sigma}_{o,i}^{-1})^{-1} \tag{2}$$

According to hypothesis 3 (see Hypotheses section), the observation uncertainty is proportional to the distance d_i: $\sigma_{o,i} = s \cdot d_i$ in the one-dimensional case (where s is a factor modelling how sensory uncertainty depends on distance). In the two-dimensional case, the uncertainty depends on the distances to the landmark in the x and y dimensions, $d_{x,i}$ and $d_{y,i}$, as well as the factors s_x and s_y controlling the dependences of the sensory uncertainties in the x and y dimensions, and the correlation ρ between x and y (see [3, 4] for more complex sensory uncertainty models).

$$\boldsymbol{\Sigma}_{o,i} = \begin{bmatrix} (s_x d_{x,i})^2 & (\rho s_x s_y d_{x,i} d_{y,i}) \\ (\rho s_y s_x d_{y,i} d_{x,i}) & (s_y d_{y,i})^2 \end{bmatrix} \tag{3}$$

Thus, the covariance matrix for the 'best guess' location estimate can be calculated from the distance measurements $d_{x,i}$ and $d_{y,i}$ to each landmark from equations (2) and (3). The covariance matrix modelling path integration uncertainty, $\boldsymbol{\Sigma}_p$, and the factors modelling the sensory uncertainty, s_x and s_y (i.e. controlling how rapidly the accuracy of distance judgements decrease with increasing distances in the x and y dimensions) and the correlation ρ are adjustable parameters.

References

1. Bromiley P (2003) Products and convolutions of Gaussian distributions. Medical School, Univ Manchester, Manchester, UK, Tech Rep 3: 2003.

2. Wu J (2004). Some Properties of the Gaussian Distribution.

3. Negenborn R (2003) Robot localization and Kalman filters. Ph.D. thesis, Utrecht University.

4. Thrun S, Burgard W, Fox D (2005) Probabilistic Robotics (Intelligent Robotics and Autonomous Agents series). Intelligent robotics and autonomous agents. The MIT Press.
