# Supplementary material for: Bayesian Integration of Information in Hippocampal Place Cells
Source: PLoS One. 2014 Mar 6;9(3):e89762. doi: 10.1371/journal.pone.0089762 (PMC3945610; doi:10.1371/journal.pone.0089762)
Supplement: Text S2 — Coincidence detection as rejection sampling and multiplication by coincidence detection. (PDF) [file pone.0089762.s002.pdf]

S2. Coincidence detection as rejection sampling and multiplication by coincidence detection

Coincidence detection as rejection sampling

In the simple three-neuron example shown in Figure 6, the computation performed by the posterior neuron (place cell), taking as inputs a prior (grid cell) and an observation (BVC), can be shown to approximate Bayesian inference (i.e. to implement equation (1) of the main text). Let us consider a temporally constrained spike train, and view each spike within this spike train as a sample taken from a probability distribution - either the spikes of the place cell, sampling the posterior location distribution $p(x|o)$, or those of the grid cell, sampling the prior location distribution $p(x)$, or the spikes of the BVC, sampling the observation distribution $p(o|x)$. In this case, the computation performed by the place cell is equivalent to the rejection sampling technique [1, 2] used to approximate an unknown distribution. In rejection sampling, in order to approximate an unknown distribution p_u, a known distribution q is sampled which satisfies

$$\forall z : p_u(z) < Mq(z) \tag{1}$$

Where $M > 1$ bounds $p_u(z)/q(z)$. To ensure that the samples approximate $p(z)$, the known $q(z)$ is iteratively sampled, and the samples are accepted with a probability proportional to the ratio

$$p_A = p(accept|z) = \frac{p_u(z)}{Mq(z)} \tag{2}$$

If each sample is randomly drawn from q, and is accepted with probability p_A, it is straightforward to show that these samples will approximate $p(z)$ [1, 2]. Briefly, the probability distribution over the accepted samples $p(z|accept)$ has to equal the unknown distribution p_u when using an infinite number of samples for the following reason. Using Bayes' theorem,

$$p(z|accept) = \frac{p(accept|z)p(z)}{p(accept)} \tag{3}$$

Where

$$p(accept|z) = \frac{p_u(z)}{Mq(z)} \tag{4}$$

And the prior probability of a sample z is given by q: $p(z) = q(z)$. The prior probability of acceptance is given by marginalizing

$$p(accept) = \int_z p(accept|z)p(z)dz = \int_z \frac{p_u(z)}{Mq(z)}q(z)dz = \frac{1}{M}\int_z p_u(z)dz = \frac{1}{M} \tag{5}$$

Thus, substituting into equation (3), we obtain the required equality.

$$p(z|accept) = \frac{\frac{p_u(z)}{Mq(z)}q(z)}{\frac{1}{M}} = p_u(z) \tag{6}$$

In our coincidence detection model (Figure 6), the acceptance probability of spikes generated by the grid cell is proportional to the spiking probability of the BVC. This is just the acceptance probability required in order to approximate a Bayesian posterior by rejection sampling, which can be shown as follows. We use the Bayesian posterior as the unknown distribution that is to be approximated (cf. equation (1) in the main text)

$$p_u = p(x|o) = \gamma p(x)p(o|x) \tag{7}$$

Where γ is a constant for normalization. We also assume that the grid cell in Figure 6 represents the prior $p(x)$ and that the BVC represents the observation likelihood $p(o|x)$. Substituting these expressions into equation (2), the acceptance probability then becomes

$$p_A = \frac{p_u(z)}{Mq(z)} = \frac{\gamma p(x)p(o|x)}{Mp(x)} = kp(o|x) \tag{8}$$

Where $k = \frac{\gamma}{M}$. Accepting samples drawn from the prior with this probability p_A ensures that the accepted samples will approximate the Bayesian posterior. Since in the simple network of Figure 6, the acceptance probability of spikes generated by the prior neuron (grid cell) is proportional to the spiking probability of the observation neuron (BVC) because of coincidence detection [3], as in equation (8) - with spiking probability 1, every grid cell spike would be coincident with a BVC spike and thus accepted, and with spiking probability 0, no grid spike would be accepted - the network approximates a Bayesian posterior, just like a rejection sampling algorithm. With infinite firing rates, the network would yield the exact posterior. In realistic cases the errors depend on the membrane time constant and firing threshold (they lie around 5% for the parameters of CA1 place cells - see Results section in the main text). Figure 7 in the main text shows the error rates of an integrate-and-fire spiking neuron with different parameters.

Multiplication by coincidence detection

This section describes a mathematical model of how coincidence detection in spiking neurons can implement multiplication with any number of inputs, and thus approximate a Bayesian posterior from multiple observations (i.e. implement equation (2) from the main text). In the following we will assume that the spatially localized firing behaviour of place cells can be approximated and modelled by probability distributions, cf. hypothesis 1 in the Introduction of the main paper.

Specifically, we will assume that place cell instantaneous firing rates are proportional to the probability density function representing the probability that the rat is in a particular location: $r \propto p$. In the one-dimensional case, the probability P_{x_A,x_B} that a rat is located on a path lying between the points x_A and x_B in the environment, which it traverses between times t_A and t_B (at which it is at locations x_A and x_B respectively), is proportional to

$$P_{x_A,x_B} = \int_{x_A}^{x_B} p(x)\,\mathrm{d}x \propto \int_{t_A}^{t_B} r(t)\,\mathrm{d}t \tag{9}$$

If the place cell does not fire, the integral on the right yields zero, which under our assumption means that the probability that the rat is in the location represented by the place cell is zero. On the other hand, if the firing rate of the place cell is very high in the time interval during which the rat crosses the place cells represented location, the probability that the rat is in that location is also very high.

One way to approximate integrals with a number of samples randomly drawn from the function to be integrated is called Monte Carlo integration [4]. If we view individual spikes of a neuron as such samples, then the density of a spike train can be viewed as approximating an integral of the form above (Monte Carlo approximation of probability distributions by spiking neurons has been suggested before, see e.g. [5–8]).

Using a binary function S to represent a spike train, which at time t is $S(t) = 1$ if a neuron has fired a spike within the time interval $[t, t+\tau)$, and 0 otherwise, equation (9) can be approximated by the spike train S as follows.

$$\int_{t_A}^{t_B} r\,\mathrm{d}t \approx \frac{1}{N} \sum_{t \in T_{A,B}} S(t) \tag{10}$$

$$P_{x_A,x_B} \propto \frac{1}{N} \sum_{t \in T_{A,B}} S(t) \tag{11}$$

Where $T_{A,B}$ denotes the interval between t_A and t_B (during which the rat was located between x_A and x_B) in τ time steps, and $N = \frac{t_b - t_a}{\tau}$ is the number of time steps of duration τ within the interval $T_{A,B}$. In the context of this paper, we can neglect multiplicative constants and work with the proportionality relations, because the most important task of localization is to find the 'best guess' location $\boldsymbol{x}_b$ in the environment, i.e. the expected value of the location $\boldsymbol{x}$, for which the amplitude of the firing rate distribution is unimportant. Finding the maximum can be expressed as in equation (12). There is an alternative and possibly more accurate [9] way of deriving a 'best guess' location from place cell firing, based on theta phase instead of the maximum firing rate (see Discussion), but that way of estimating location does not depend on absolute firing rates either. The 'best guess' location $\boldsymbol{x}_b$ based on the expected value of the location distribution can be calculated from the function $S(t)$ representing the spike train, the locations $\boldsymbol{x}(t)$ at the time of each spike, and the total number of spikes K in this interval, which is the sum of $S(t)$ over $T_{A,B}$:

$$\boldsymbol{x}_b = \mathbb{E}[\boldsymbol{x}] \approx \frac{1}{K} \sum_{t \in T_{A,B}} S(t)\boldsymbol{x}(t) = \frac{\sum_{t \in T_{A,B}} S(t)\boldsymbol{x}(t)}{\sum_{t \in T_{A,B}} S(t)} \tag{12}$$

The number of spikes is $K = \sum_{t \in T_{A,B}} S(t)$. Using standard deviation as a measure of uncertainty, the location uncertainty can be described as

$$\boldsymbol{\sigma}_b = \sqrt{\mathrm{Var}(p)} \approx \sqrt{\frac{1}{K} \sum_{t \in T_{A,B}} (S(t)\boldsymbol{x}(t) - \boldsymbol{x}_b)^2} \tag{13}$$

Using this way of approximating probability distribution functions with spike train densities, coincidence detection in spiking neurons can implement multiplication. Looking at two neurons A and B providing input to a third neuron C which performs coincidence detection, it can be shown that the function represented by C's spike train will approximate the product of the functions approximated by A and B. If we set the time discretization parameter τ, to the temporal resolution of coincidence detection (which mainly depends on the membrane potential and signal-to-noise ratio, see [10] or Figure 7 in the Results for an error analysis), and ensure that C's spike threshold is high enough so that C only fires if input spikes arrive from both A and B within τ, then the function represented by C's spike train S_C will depend on the product of S_A and S_B:

$$S_C(t) = S_A(t)S_B(t) \tag{14}$$

Equation (14) is also extensible to the multiplication of a larger number M of input neurons Ni. If the threshold of the output neuron N is so high as to require synchronous spikes from *all* inputs within a time τ, it can simply be extended to calculate the function S_O representing the spike train of the output neuron:

$$S_O(t) = \prod_{i}^{M} S_{Ni}(t) \tag{15}$$

In general, the threshold will not be so high as equation (15) assumes. Hence, the output neuron will generally have a threshold such that a proportion $\alpha = (0, 1]$ of all input neurons M spiking synchronously can elicit an output spike (i.e. there is an output spike only if $m > M\alpha$ input spikes arrive within τ). Then the approximation of a product of multiple input spike trains becomes

$$S_O(t) = \mathrm{H}\left(\frac{1}{M}\sum_{i=1}^{M}(S_i(t) - \alpha)\right) \tag{16}$$

Where $\mathrm{H}(a) = \begin{cases} 0 & \text{if } a < 0 \\ 1 & \text{if } a \geq 0 \end{cases}$ is the Heaviside step function. This equation equals (15) if $\alpha = 1$, and approximates it otherwise. It is a simplified formulation of how coincidence detection can perform multiplication in a spiking neuron. We can insert it into equation (11) to obtain the approximation of a probability distribution as follows.

$$P_{x_A,x_B} \propto \frac{1}{N}\sum_{t \in T_{A,B}} S_O(t) \tag{17}$$

The expected value of this function, i.e. the represented 'best guess' location, will approximate the mean of the product of input functions - see equation (12).

$$\boldsymbol{x}_b = \mathbb{E}[\boldsymbol{x}] \approx \frac{1}{K}\sum_{t \in T_{A,B}} S_O(t)\boldsymbol{x}(t) = \frac{\sum_{t \in T_{A,B}} S_O(t)\boldsymbol{x}(t)}{\sum_{t \in T_{A,B}} S_O(t)} \tag{18}$$

The particular threshold of the output neuron plays a large role in determining the accuracy of this approximation, as do the number of samples (spikes). This computation requires the neuronal parameters influencing temporal resolution and the threshold to be within a certain range to allow for reasonably accurate localization. Our computational simulations indicate that the empirically observed parameters of hippocampal place cells are indeed within a range to allow for statistically near-optimal localization (see the Results section in the main text).

References

1. Liu JS (1996) Metropolized independent sampling with comparisons to rejection sampling and importance sampling. Statistics and Computing 6: 113–119.

2. Bishop CM, et al. (2006) Pattern recognition and machine learning, volume 4. springer New York.

3. Rossant C, Leijon S, Magnusson A, Brette R (2011) Sensitivity of noisy neurons to coincident inputs. The Journal of Neuroscience 31: 17193–17206.

4. Robert CP, Casella G (1999) Monte Carlo Statistical Methods. Springer-Verlag, 1 edition. URL `http://www.amazon.com/exec/obidos/redirect?tag=citeulike07-20&path=ASIN/038798707X`.

5. Hoyer PO, Hyvärinen A (2003) Interpreting neural response variability as Monte Carlo sampling of the posterior, MIT Press, volume 15. p. 293.

6. Paulin MG (2005) Evolution of the cerebellum as a neuronal machine for Bayesian state estimation. Journal of Neural Engineering 2: S219–S234.

7. Paulin MG, Hoffman LF (2011) Bayesian head state prediction: Computing the dynamic prior with spiking neurons. 2011 Seventh International Conference on Natural Computation : 445–449.

8. Büsing L, Bill J, Nessler B, Maass W (2011) Neural dynamics as sampling: a model for stochastic computation in recurrent networks of spiking neurons. PLoS Computational Biology 7: e1002211.

9. Jensen O, Lisman JE (2000) Position reconstruction from an ensemble of hippocampal place cells: contribution of theta phase coding. Journal of Neurophysiology 83: 2602–2609.

10. Brette R (2012) Computing with neural synchrony. PLoS Computational Biology 8: e1002561.
